# Effectiveness of diabetes education and awareness of diabetes mellitus in combating diabetes in the United Kigdom; a literature review

**Published:** 2015-09-09

**Authors:** Chaudhary Muhammad Junaid Nazar, Micheal Mauton Bojerenu, Muhammad Safdar, Jibran Marwat

**Affiliations:** ^1^Department of Nephrology, Shifa International Hospital, Islamabad, Pakistan; ^2^Department of Internal Medicine, Sickle Cell Unit, Harvard University Hospital, Washington DC, USA

**Keywords:** Diabetes mellitus, Prevalence, Glycosylated hemoglobin, Education

## Abstract

Diabetes mellitus is a metabolic disorder that is characterized by high blood glucose level, and body cannot produce enough insulin, or does not respond to the produced insulin. In spite of the diabetes education campaigns and programmes, a large number of people in the United Kingdom are living with diabetes. The main objective of the study is to evaluate the role of knowledge and awareness of diabetes in fighting against diabetes and to interpret to which extent is diabetes education successful. The systematic review to be carried out will include literature from 2001 to 2011 in the United Kingdom regarding awareness of diabetes among UK population and effectiveness of diabetes education. Literature will be accessed using search database, British medical journals, and library. Good quality papers will be used for the systematic review. Previous studies about diabetes education will consulted and assessed. This study is going to summarize the efficacy of diabetes education campaigns and programmes which are promising to enhance the awareness The outcome of the review will be the guideline for the government, education centres, researchers, and campaigns to implement more diabetic education programmes and easily accessible diabetes services and education interventions to increase the awareness of risk factors and complications of diabetes to overcome the increasing epidemic of diabetes in the United Kingdom.

**Implication for health policy/practice/research/medical education:**

Diabetes mellitus is a metabolic disorder, in which there is high blood glucose level, and body cannot produce enough insulin, or the body does not respond to the insulin produced. In spite of the diabetes education campaigns and programmes, a large number of people in the United Kingdom are living with diabetes. The main objective of the study is to evaluate the role of knowledge and awareness of diabetes in fighting against diabetes and to interpret to which extent is diabetes education successful. The outcome of the review will be the guideline for the government, education centres, researchers, and campaigns to implement more diabetic education programmes and easily accessible diabetes services and education interventions to increase the awareness of risk factors and complications of diabetes to overcome the increasing epidemic of diabetes in in the United Kingdom.

## Introduction

Diabetes is a serious and life-threatening disease, however it can be managed very well through proper treatment and controlling. Diabetes self-management training and education plays a vital role in the management of diabetes (1). It is crucial for diabetic patients to be aware of nature, treatment, risk factors and complication of disease due to providing suitable modality to attenuate following complications. In a study to detect the relation between health literacy, complication awareness and diabetic control among patients with type 2 diabetes mellitus, it was concluded that patient awareness scores and health literacy was negatively related to diabetes control (2). This study was 6 months study, carried out from September 2005 to February 2006 with about 150 Chinese patients.

## Materials and Methods

For this review, we used a variety of sources by searching through PubMed, EMBASE, Scopus and directory of open access journals (DOAJ). The search was performed by using combinations of the following key words and or their equivalents; Prevalence of diabetes mellitus, awareness and knowledge about diabetes and its management, diabetes education programmes, effectiveness of diabetes education.

## Results

Looking at the study carried out to explore the total prevalence of diabetes mellitus in 2001 in England to support delivery of healthcare services it was estimated that in 2001 the prevalence of diabetes (diagnosed as well as undiagnosed) in England was about 4.5%, affecting more than 2 million persons (3). It was found that the prevalence of type 2 diabetes was 92% affecting 2000000 persons and the prevalence of type 1 diabetes was nearly 8% affecting 160000 persons. The prevalence of diabetes was estimated to be more in women (5.2%) than men (3.6%). It was also estimated that the prevalence of diabetes was higher in the people from ethnic minority groups than the white people. The estimated prevalence rates are 4.3 for white people, 5.7 for black African/Caribbean, and 6.6 for South Asians and 2.1% for other groups. The prevalence of diabetes was found to be increased rapidly with age as the prevalence was found to be 0.3 in people aged 0–29, 3.3 in those 30–59 and 14% in people over 60 years age.

According to Diabetes UK (2010) in 2009, the prevalence of diabetes in adults over 17 years old is estimated to be 5.1% in England affecting 2213138, 4.5% in Northern Ireland affecting 65066, 4.6% in Wales affecting 146173 and 3.9% in Scotland affecting 209886 people. The total average prevalence of diabetes in 2009 in the United Kingdom is estimated to be 4.26%.

A systematic review was conducted to estimate the age- and sex-specific diabetes prevalence worldwide for years 2010 and 2030 (4). Studies from 91 countries were selected and it was found from the review findings that the incidence of diabetes among people aged 20–80 years will be 6.5% in 2010 and 286 million adults will be affected in 2010. The prevalence of diabetes will increase to 7.8%, and nearly 440 million adults will be affected by 2030. It was suggested that there will be a 70% increase in the prevalence of diabetes in adults of developing countries and about 21% rise in developed countries. By looking at CHASE study, a cross-sectional survey carried out involving nearly 4800 children aged 9-10 years old recruited from London, Birmingham and Leicester, it is found that South Asians adults, residents of UK are 3 times more prone to develop type 2 diabetes than white Europeans (5,6). These people have higher blood levels of glycated haemoglobin (HbA1c), higher level of C-reactive proteins in the blood, lower level of High-density lipoprotein -cholesterol (HDL-C) and high triglyceride levels than white people. Black African-Caribbean adults residing in the United Kingdom have also most of these diabetic risk factors but these people have high HDL-C levels and low triglyceride levels.

Better diabetic education and knowledge to control and treat diabetes at right time can minimize the chances to develop complications of diabetes and thus reduce morbidity and mortality in diabetics (7,8). It suggests that as the rising figures of people diagnosed with diabetes is becoming a challenge in the United Kingdom so a randomised clinical trial will be run by independent research teams to interpret effective delivery and cost effectiveness of CASCADE (Child and Adolescent Structured Competencies Approach to Diabetes Education) for children and young people involved in this trial. As we know that if diabetes is diagnosed in childhood and bitterly controlled, the chances to develop long-term complications become less. The CASCADE is a multi-centre randomised control trial involving 26 clinics randomly selected as control/intervention groups, including 572 children and young people (7). Despite of the advanced medications and their delivery systems there is less improvement in control of diabetes in children and young people in the United Kingdom in last decade (8). So new health delivery systems are needed for children and young people to improve and control the diabetes.

With regards to this, in 2010, fifth national survey was carried out to assess the delivery of UK diabetes services to children and young people and identified changes in service delivery systems since 2002 (9). One hundred twenty-nine services took part in the survey involving 220 clinics. Ninety-eight percent of paediatric consultants were found having special interest in diabetes whereas in 2002 about 89% of consultants were interested in diabetes. In 88% of services, the diabetes specialist nurse worked alone in paediatric diabetes compared to 53% of the services in 2002. So overall it was concluded that there is much improvement in diabetes services for children providing high quality care, but serious deficiencies still remains.

According to Diabetes UK (2010) most of the people with diabetes type 2 in the United Kingdom are over 60; their level of diabetes knowledge tends to be poorer. According to Diabetes UK (2010) report, the residents of care homes fail to receive diabetes education and screening. A care home resident gets admitted to the hospital for screening and diagnosis of diabetes due to the lack of screening facilities and lack of diabetes education. There are diabetic residents in 6 out of 10 care homes that cannot provide special education (10).

UK prospective diabetes study has shown that adapting the effective therapy to reduce high blood pressure and high blood glucose level will result in reducing the diabetes complications (11). Diabetes UK invested more than 2 million on this study (11). The UK Prospective Diabetes Study, the 20-year study involving 5000 patients with diabetes in the United Kingdom, has revealed that intensive blood glucose level control and adopting better treatment methods can reduce the risk of diabetic retinopathy by a quarter and early renal damage by a third (11). Intensive management and control of blood pressure in hypertensive patients can reduce the risk of death resulting from life threatening long-term complications of diabetes by a third, vision loss by more than a third and cardiovascular disease by more than a third (10).

By looking at the data collected between 1st April 2008 and 31st March 2010 from 1421 weight reducing operations carried out, it is found that before surgery 379 of these 1421 patients were having type 2 diabetes (11). After 1 year of surgery it was found that this number of diabetic patients was decreased to 188 from 379 (11). Therefore by providing knowledge of advance treatment methods to people helps in controlling the diabetes as educating people about the weight loss surgeries (gastric bypass and gastric bands) can tackle type 2 diabetes as seen in this study.

Diabetes education can improve the quality of life of diabetic patients and can also prevent the costs of long-term complications of diabetes in the patients (10). As amputation of lower limb in a diabetic patient, a long-term complication of diabetes is a costly intervention, the diabetes education can help in reducing the amputation rate that can lead to large cost savings (10). Diabetic foot ulcers can develop in patients having diabetes both in type 1 and type 2 diabetes (11). It has been found, 10% of diabetic individuals may suffer from foot ulcer during their lifetime. Foot ulcer often occurs in the people who develop peripheral diabetic neuropathy and also by wearing tight shoes, by walking on tread mill, having cuts, blisters and also having narrowed arteries; atherosclerotic peripheral arterial disease. The diabetic foot ulcers should not be avoided and diabetic foot needs a special care, otherwise the diabetic foot ulcer can result in the amputation of the foot even the whole lower limb (11). The risk of lower limb amputation in diabetic patients is 15 to 45 times more than in people with no diabetes (10). About 25% of hospital admissions of diabetic people in United States and Great Britain are due to diabetic foot complications (10). The annual incidence of diabetic foot ulcers and amputation are 2.5% to 10.7% and 0.25% to 1.8%, respectively (12).

In the United States an estimated more than 130 billion dollars in 2002 is the cost of diabetes (13). Because of these devastating numbers, the cost-efficacy of preventing and treating diabetes, and the cost-effectiveness of diabetes self-management training and medical nutrition therapy to treat diabetes are receiving much attention (13). While in the United Kingdom, the cost of diabetes to the National Health Service (NHS) stands at approximately £1 million per hour, and is increasing rapidly. Diabetes accounts for approximately a tenth of NHS budget each year, a total exceeding £9 billion (11). With regards to this a systematic review was carried out involving 26 articles including randomized controlled trials, retrospective database analyses, meta-analysis, prospective, quasi-experimental and, to evaluate the cost-effectiveness of diabetes education. The results of more than half of the studies reviewed were indicated positive association between diabetes education and decreased cost. The findings of these studies indicate that diabetes self-management education (DSME) has more benefits in reducing the costs associated with diabetes intervention. Study agreed with this finding by conducting a 12-month study involving primary care trusts in the United Kingdom to assess the long-term clinical and cost-effectiveness of the diabetes education and self-management for ongoing and newly diagnosed (DESMOND) intervention (14). The cost-utility analysis was undertaken using data from a 12-month, multicentre, cluster randomised controlled trial and the study resulted that the DESMOND intervention is considered to be cost effective compared with usual care, especially with respect to the real world cost of the intervention to primary care trusts, with reductions in Cardiovascular disease (CVD) risk especially reduction in weight and smoking (14).

According to a cohort study, conducted in 2005 by Diabetes UK, The cancer risk and mortality is progressively elevating in insulin treated diabetic individuals (15). This study involved 28900 UK resident patients with insulin-treated diabetes who were less than 50 years old at the diagnosis of diabetes. However, the results showed, risks of some cancers such as liver, pancreatic, endometrial, renal and colorectal cancer slightly are raising in patients with prime type 2 diabetes but some cancer incidence including gall bladder, breast cancers and non-Hodgkin lymphoma (NHL) have not changed or prostate cancer risk has been reduced (15).

Celiac disease, as a chronic immune mediated disorder, is triggered by gluten intake in predisposed patients (16). Type 1 diabetes is one of the diseases associated with celiac disease (18). Both diseases have a common genetic predisposition. In one Turkish study involving 100 diabetic patients (51 female, 49 male, mean age 26 ±9 years, and 80 control subjects - 40 female, 40 male, mean age 27 ± 8 years), it was estimated that the prevalence of celiac disease is more in diabetic patients than the general people and celiac disease in diabetic patients can only be diagnosed by screening tests for celiac disease as CD is mostly seen as asymptomatic in these patients. The most sensitive and specific test for the diagnosis of CD is the anti-endomysial IgA antibody (IgA-EMA) test with a sensitivity of more than 90% and a specificity about 100%. This is a screening method in patients at high risk for CD. Anti-endomysium IgA was tested by indirect immunofluorescence using sections of human umbilical cord for screening. Some investigators predicted that the complications of diabetes are increased in the presence of celiac disease and worsens the metabolic control in these diabetic patients (17).

High blood glucose level can lead to microvascular and macrovascular complications (18). For examining this, a prospective observational study (UKPDS 35) was conducted by Stratton et al (18). To report positive correlation between hyperglycaemia and macro/micro-vascular insults in type 2 diabetic patients. This study involved 23 hospital-based clinics in England, Scotland and Northern Ireland. About 4600 patients including white, Asian Indian and African-Caribbean patients were participated in incidence rates analysis. Risk factors related macro-vascular complication were noticed in about 3600 of the total patient. The results of the study indicated that there is a direct relation between hyperglycemia, micro-vascular and macro-vascular complications (18). This is also clear by examining a cohort study, conducted by Fuller et al to assess cardiovascular disease associated risk in type 1 diabetic patients in the United Kingdom (19). This study consisted of group of 7500 patients with type 1 diabetes and 5 age- and gender-matched controls per non-diabetic individuals comparison group (nearly 38200) selected from the General Practice Research Database (GPRD). The cardiovascular events in these two groups were apprehended between1992-1999. These high CVD risks were seen for strokes, acute coronary disorders, and for coronary revascularizations. Results showed that women having type 1 diabetes continue to experience greater relative risks of cardiovascular disease than men compared with those without diabetes (19). Hence, there is increased absolute and relative risk of mortality due to CVD in patients with type 1diabetes compared with those without diabetes in the United Kingdom (19).

Blood glucose awareness training and cognitive behavioural therapy have been able to balance blood glucose level in type 1 diabetic patients (20). To support this evidence, a systematic review was completed (20) in Oxford to assess fear of hypoglycaemia in the patients having diabetes. About 36 papers were reviewed. And it was implicated from the review that fear of hypoglycaemia can have negative impact on diabetes management and awareness training is needed to reduce this fear of hypoglycaemia. This was further supported by a randomised control trial, carried out (21) on 650 randomly selected diabetic patients from Bournemouth Diabetes and Endocrine Centre’s diabetes register to determine the relationship between numeracy skills and glycaemic control in type 1 diabetes. Out of 650 patients 112 patients completed the study. Forty-seven percent were the male patients and it was found that low numeracy skills were badly associated with glycaemic control in diabetes and literacy was also badly associated with glycaemic control in diabetes and also relationship between literacy and glycaemic control was found to be independent of the duration of diabetes and socio-economic status of the patients.

Diabetic patients can develop hyperglycaemia and hypoglycaemia in the critical care setting while hospitalized due to various factors including infection, poor diet, and drugs (22). Hospitalized patients can develop hyperglycaemia even in the absence of family history of diabetes (22). The blood glucose level range of 100–200 mg/dl is the target of glycaemic control in the hospitalized patients. Insulin infusion is done in hospitalized patients having type 1 diabetes and in type 2 diabetic patients, oral drugs are stopped and insulin is started for glycaemic control (22).

Educational and psychosocial interventions are able to approximately improve diabetes management. (23,24). A systematic review was completed by Hampson et al (23) to investigate the educational and psychosocial intervention efficacy on improvement of diabetes management in adolescents type 1 diabetes patients. About 60 articles were reviewed. This systematic review gave the result that educational and psychosocial interventions have beneficial impacts on various diabetes management consequences. Similarly a systematic review was conducted by Norris et al (24) to assess the effectiveness of self-management education on glycosylated hemoglobin in adults having type 2 diabetes. Total 31 articles on randomized control trials were reviewed and it was found that DSME improves glycated hemoglobin levels at immediate follow-up by 0.76%, that long-lasting interventions may be needed to maintain the improved glycaemic control brought about by DSME programs as the more contact time between patient and educator enhances the efficacy of the result and that the improvement in glycosylated hemoglobin level drops 1–3 months after the intervention ceases (24). Further supporting this, another systematic review was conducted by Hawthorne et al (25) to determine the efficacy of various diabetic diet advice on balancing blood glucose level and weight in type 2 diabetic individuals. Only randomized controlled trials of 6 months or longer, were selected for the review and total 36 articles were reviewed. In this review study, some parameters such as weight, mortality, maximal exercise capacity and compliance various lipoproteins levels and blood pressure were measured. The review indicated that dietary advice is effective in the glycaemic control in type 2 diabetes mellitus (25) further supported all these reviews by conducting a systematic review to assess the effectiveness of culturally appropriate diabetes health education on type 2 diabetes mellitus as prevalence of type 2 diabetes mellitus is higher in ethnic minorities in the developed countries like the United Kingdom (25). Eleven randomised control trials of culturally appropriate diabetes health education on people having type 2 diabetes over 15 years from defined ethnic minority groups of developed countries were reviewed. The trials indicated both glycaemic control as well as improvement in knowledge after culturally appropriate diabetes education interventions. It was suggested from the review that culturally appropriate diabetes health education is effective in glycaemic control in type 2 diabetes and improving the knowledge score and changing the lifestyles and attitudes of the people.

## Conclusion

Various diabetes education courses are being carried out in the United Kingdom, including DAFNE, DESMOND and X-PERT in order to increase awareness and knowledge of diabetes among people. These diabetes courses are designed to empower diabetic patients to manage their own condition effectively. Various factors like cost, distance, shortage of enough educators or centres, lack of appropriate services affect many people with diabetes to get access to diabetes knowledge. Educating the patients regarding diabetes have a key role in encouraging and supporting them to assume active responsibility for the day to day control of their situation. The review depicts that illiteracy and lack of knowledge poses a great challenge to effective health education. The review demonstrates that south Asian patients face problems regarding diet aspect and show poor level of knowledge about diabetes and also are discouraged to join educational sessions. The review indicates that impaired awareness of the diabetes increases the chances to develop complications of diabetes as the severe hypoglycaemia is becoming more common in insulin treated type 2 diabetes than previously recognized and with increased duration of insulin therapy may increase to meet that observed in type 1 diabetes. The risk of severe hypoglycaemia increases with having impaired awareness of hypoglycaemia. The authors has concluded that diabetes associated complications and psychological insults is usual in diabetic individuals. The study indicates that many providers involved in the study are aware of the diabetes related psychological problems but lack confidence in their ability to evaluate these problems and to support these patients. So, there is a need for manipulating models of care that provide essential psychosocial services. There is also need of integrating mental health professionals into the diabetes care team. This study will help the government to implement the diabetes education programmes that are cost effective and attractive to the public, easy to get access. Any diabetes service should provide highly structured diabetes education programme. In spite of the advanced medications and their delivery systems there is less improvement in control of diabetes in children and young people in UK in last decade. Better diabetic education and knowledge to control and treat diabetes at right time can reduce the risk factors and minimize the chances to develop complications of diabetes and thus reduce morbidity and mortality in diabetics.

## Authors’ contribution

CMJN completed the article, MS and MMB reviewed the article, and JM completed the draft.

## Conflicts of interest

The authors declared no competing interests.

## Ethical considerations

Ethical issues (including plagiarism, data fabrication, double publication) have been completely observed by the authors.

## Funding/Support

None.
